# Safety and Efficacy of Radiofrequency Ablation and Epidural Steroid Injection for Management of Cervicogenic Headaches and Neck Pain: Meta-Analysis and Literature Review

**DOI:** 10.7759/cureus.34932

**Published:** 2023-02-13

**Authors:** Chukwuyem Ekhator, Alyssa Urbi, Basil N Nduma, Solomon Ambe, Ekokobe Fonkem

**Affiliations:** 1 Neuro-Oncology, New York Institute of Technology, College of Osteopathic Medicine, Old Westbury, USA; 2 Neuro-Oncology, Brandeis University, Boston, USA; 3 Internal Medicine, Merit Health Wesley, Hattiesburg, USA; 4 Neurology, Baylor Scott and White Health, Mckinney, USA; 5 Neuro-Oncology, Baylor Scott and White Health, Temple, USA

**Keywords:** headache, neck pain, epidural steroid injection, radio-frequency ablation, cervicogenic headache

## Abstract

Dysfunction of the cervical spine and its anatomical features, mostly innervated by the C1, C2, and C3 spinal nerves, can result in a secondary headache known as cervicogenic headache (CHA), mainly characterized by unilateral pain. The usefulness of pharmaceutical medications and physical therapy is currently the subject of scant literature. Interventional pain management techniques can be applied when conservative treatment is unsuccessful. This study looks at radiofrequency ablation (RFA) and epidural steroid injection (ESI) to identify their safety and efficacy in managing patients with cervicogenic headaches and neck pain. Three databases - PubMed, Cochrane CENTRAL Library, and Embase were searched, and 110 studies were identified. Nine screening processes were included for review and meta-analysis. Statistical evaluation was conducted through STATA version 17 (College Station, TX: StataCorp LLC) and effect measures were reported through random effects model risk ratios. The main subject of focus included three following outcomes: incidences of pain relief, degree and duration of pain, and incidences of adverse effects. The findings showed both interventions relieved pain by a factor of >50%, demonstrating a relative effects risk ratio of 1.45 (-0.50, 3.39) for RFA: pain relief, 84.76 (82.82, 86.69) RFA: adverse effects, and 19.46 (18.80, 20.11) ESI: pain relief at 95% confidence interval. The efficacy of RFA and ESI differ. Both interventions are effective in the reduction of cervicogenic headache pain intensity. However, their complication rates and pain duration are considerably different. With ESI, the headaches can still recur weekly, demanding the use of oral analgesics to deal with them. On the other hand, RFA has a low complication rate. Improving guidance from imaging technologies, RFA has the potential to be the most effective interventional treatment.

## Introduction and background

Dysfunction of the cervical spine and its anatomical features, mostly innervated by the C1, C2, and C3 spinal nerves, can result in a secondary headache known as cervicogenic headache (CHA), characterized by unilateral pain [1]. CHA is challenging to identify and manage due to its substantial overlap with migraine headaches and the lack of readily available testing and diagnostic criteria [2]. There is no obvious male or female predominance in the occurrence of CHA in the general population, of which those with CHA ranges from 1% to 4.1% [3]. The convergence of nociceptive afferents from the trigeminal and upper three cervical nerves onto the second-order neurons in the trigeminocervical nucleus in the upper cervical spinal cord (C1-C3) is the etiology of CHA. Therefore, the origin of CHA is implicated in every cervical component innervated by the joints, muscles, nerves, ligaments, and dura [4]. The average age of onset is 43 years. Nausea, vomiting, and throbbing pain are common signs of the syndrome [5]. The presence of a mechanical etiology is frequently related to CHA but is not necessary for diagnosis. Although computed tomography (CT), myelography, and magnetic resonance imaging (MRI) can help support the diagnosis, these imaging procedures are frequently more helpful in excluding secondary causes [6]. A history and physical examination are the best methods for identifying this syndrome and excluding other systemic diseases [7].

Candidates for injection therapies are amongst those who have not responded to more conservative forms of treatment such as activity restriction, manual and physical therapy, and oral or transdermal pharmaceutical trials [8]. Interventional pain management techniques can be either therapeutic or diagnostic. Risks related to infection, radiation exposure, corticosteroid side effects, and structural damage from spinal needle implantation are all part of interventional pain management techniques. Despite the possibility of a problem where the needle contacts a traversing nerve root while passing through joint, Z-joint injections are generally viewed as being rather safe operations because the needle is accessing parts of the spine outside of the spinal canal [9]. When administering lateral joint injections, extra care must be taken to avoid damaging the vertebral artery or the C2 spinal nerve.

There have been substantial developments in the knowledge of the pathogenesis and treatment of chronic axial neck pain and cervicogenic headache, which are both frequent issues [10]. The intensity and length of the pain drive the process. Strengthening exercises for the anterior, posterior, and interscapular muscle groups are advised for patients who have experienced mild-to-moderate pain for less than six months and have no discernible motor loss. These exercises should also include body mechanics instruction. If the patient is doing well after eight weeks, activities can continue at home or in a gym. Physical therapy may be continued for up to another eight weeks if the patient is not getting better [11]. At the first appointment, radiographs and magnetic resonance imaging (MRI) should be requested for individuals with motor loss or significant pain. Patients with mild-to-moderate pain who do not feel better after four to six months should have neck MRIs and plain radiographs are taken. Typically, a spinal injection is advised based on the findings. An epidural corticosteroid injection should be requested if an MRI shows spinal stenosis of the central or lateral canal or a herniated disc [5]. If the epidural offers effective pain relief, the patient can be recommended for more intensive physical therapy and have the procedure repeated up to three times [5,9,11].

The most extensively studied interventional therapy for CHA is radiofrequency ablation (RFA) of the cervical medial branch with third occipital nerve (TON) neurotomy [12]. RFA of the cervical medial branch nerve and TON is used to destroy the afferent nerve supply, which is thought to be the main source of discomfort for the CHA. With monopolar RFA, a ground plate with a sizable surface area is put to the body to produce a thermal lesion. Radiofrequency (RF) ablative treatments have been widely accepted in specialties like oncology, cardiology, and chronic pain. Studies have demonstrated the effectiveness of RFA in treating some pain conditions [9,13]. It is believed that interrupting these nerves' normal activity for a small period of time with an anesthetic block or a semi-permanent period with RF lesioning causes Wallerian degeneration of the afferent nerve fibers, which frequently reduces this referred pain [5,12,13]. However, according to this study and others, pain alleviation is typically just transient and only lasts till the time of nerve regeneration or healing. While RFA alleviates the crippling pain, it does not address the headache's underlying causes, which are frequently never fully determined [12,13].

Substantial evidence exists showing that certain patients with radicular pain or radiculopathy respond favorably to cervical epidural steroid injection as a form of anesthetic treatment [9]. However, the time that pain alleviation lasts varies [7,14,15]. In order to treat several types of pain, including nociceptive pain, neuropathic pain, sympathetic-mediated pain, malignant pain, and visceral pain, corticosteroids are often utilized with local anesthetics or adrenergic blocking medications, such as guanethidine, in nerve blocks. The pain cycle can be broken by local blocks, typically reversible, and may offer long-lasting pain relief [9-11]. Both interlaminar and transforaminal routes can reach the cervical epidural space, and fluoroscopy is advised for both.

The usefulness of pharmaceutical medications and physical therapy, like muscle stretching and manual cervical traction, is currently the subject of scant literature [11]. Interventional pain management techniques can be applied when conservative treatment is unsuccessful. These include RFAs, occipital nerve blocks, cervical spinal rami blocks, cervical epidural steroid injections, and occipital nerve stimulation. Although surgical procedures are another choice, they are frequently viewed as a last resort due to inefficiency and the high risk of consequences. Finding a successful treatment is of utmost clinical significance since CHA does not get better with time, unlike other secondary headaches. Therefore, this study looks at radiofrequency ablation and epidural steroid injection to identify their safety and efficacy in managing patients with cervicogenic headaches and neck pain [16-19].

Methodology

Study Design

This is a systematic review and meta-analysis. The Preferred Reporting Items for Systematic Reviews and Meta-Analyses (PRISMA) guidelines were used to guide the execution of this study. The initial search for relevant literature was conducted from PubMed, Cochrane CENTRAL Library, and Embase. We used both keyword combinations and Medical Subject Headings (MeSH) terms. We also used Boolean operators (AND/OR) and field tags (tw/tiab) to narrow the search results. More studies were obtained through reference lists of former systematic reviews and meta-analyses on this topic. In all three databases, a detailed search was performed using conceptual keywords "radiofrequency ablation" OR "epidural steroid injection" AND "cervicogenic headache." The three keywords were coupled with the following MeSH terms: "radiofrequency ablation"(MeSH) OR "injections, epidural"(MeSH) AND "post-traumatic headache"(MeSH). When all the components were brought together, two search strings were generated. The two search strings used in the literature search are illustrated below.

The search string one using keyword concepts 1 and 3 include ("radiofrequency ablation"{MeSH Terms} OR "radiofrequency ablation*"{Text Word} OR "radiofrequency therapy"{Text Word}) AND ("post-traumatic headache"{MeSH Terms} OR "cervicogenic headache*"{Text Word} OR "chronic headache*"{Text Word}). The search string two using keyword concepts 1 and 3 include ("injections, epidural"{MeSH Terms} OR "epidural*"{Text Word} OR "steroid injection*"{Text Word}) AND ("post-traumatic headache"{MeSH Terms} OR "cervicogenic headache*"{Text Word} OR "chronic headache*"{Text Word}). A search filter for language was included to only output studies published in English. The search process described above was executed in November 2022.

Eligibility Criteria

The participants, exposures, comparators, outcomes, study designs (PECOS) were used to create inclusion and exclusion criteria for the selected studies. Participants (P) of the eligible studies were adults manifesting cervicogenic headaches, neck pains, or any post-traumatic headache. No age limit was placed on the inclusion of participants. We required the included studies to be from patients who have been exposed (E) to either of the two target interventions - radiofrequency ablation treatment or epidural steroid injection. The comparator (C) was not a consideration for this experiment. The review sought to conduct a single-arm analysis to elucidate the efficacy and safety of using either intervention to manage the aforementioned conditions. The study was focused on outcomes (O) indicating the effectiveness and safety of RFA and epidural steroid injection (ESI). Specifically, we were interested in studies reporting a reduction in pain intensity, observations on the duration of pain, and adverse effects of treatment. The criteria were relatively open on the inclusion of various study designs. An array of experimental and observational study designs was included for literal synthesis or statistical analysis.

Data Extraction and Analysis

Selected studies were forwarded to another pair of data extractors. This was conducted in a standardized Excel sheet, which encoded various variables of interest to the reviewers. First, details for study identification, such as the first author, year of publication, and the study design, were extracted. Participant demographics were also extracted, followed by details regarding the interventions. The review was interested in knowing the type of intervention (specified by having two data tables dedicated to our interventions of interest), the location of administration, and the follow-up period after administration. The outcomes of each study were recorded by first identifying which outcomes were observed, what the results of these observations were, and the dichotomous outcomes of at least one of our three outcomes of interest (reduction in pain intensity, observations on the duration of pain, and adverse effects of treatment). Data regarding participants' opinions on the application of radiofrequency ablation and epidural steroid injection were also a point of interest. The extracted data were analyzed in the following two phases: all qualitative information was synthesized thematically using literal analysis to review the selected literature. On the other hand, quantitative data were analyzed using STATA version 17.0 (STATA v.17.0; College Station, TX: StataCorp LLC) to determine each outcome's effect measure and significance. Heterogeneity between the studies was assessed using the I2 statistic. The meta-analysis adopted a random effects model to evaluate both binaries. The effect measure was the risk ratio at a 95% confidence interval. The results of the meta-analysis were represented graphically in forest and funnel plots.

## Review

Study selection

**Figure 1:**
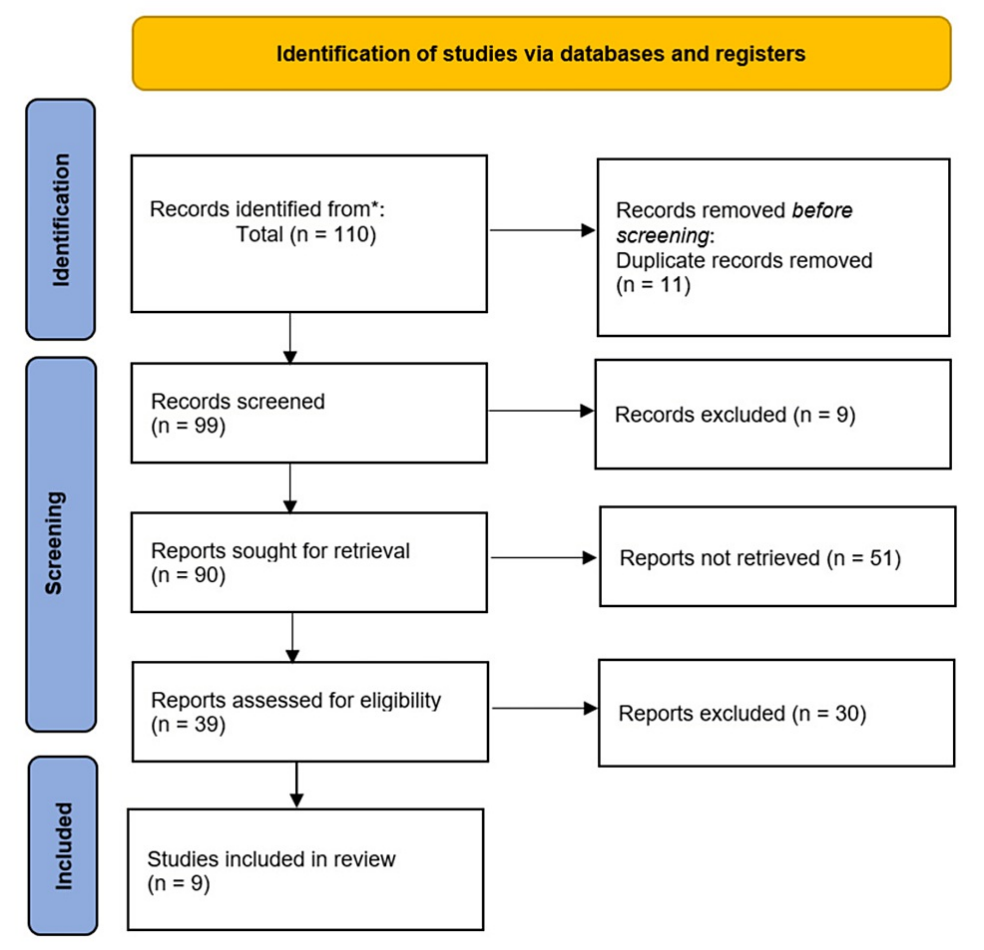
PRISMA flow diagram detailing the study selection process. *All databases.

The complete search process yielded 110 articles from all databases searched and reference lists. The selection process began by eliminating duplicates, where 11 were eliminated. The first screening process eliminated nine studies in an automated process leaving 90 studies for the title and abstract screening. In this phase, we sought to include studies assessing the right participants and exposure. A total of 51 studies were eliminated, leaving behind 39 studies. These were assessed again in the full-text screening phase to ascertain they contained the outcomes of interest and reported the data in a utilizable manner. Again, 30 studies did not pass this criterion, and we eliminated leaving behind nine for inclusion. Figure 1 below is a PRISMA flow diagram outlining the selection process.

Epidural steroid injection 

Table 1 outlines summarized characteristics of the studies included under the epidural steroid injection.

**Table 1:** Characteristics of the studies included under the epidural steroid injection.

Author	Study design	Participants	Location of injection	Follow-up period	Pain measurement tool	Outcomes observed	Results	Pain relief (>50%)	Duration of pain improvement	Incidence of adverse effects
Haspeslagh et al. (2006) [19]	RCT	Fifteen patients with cervicogenic headaches underwent local injections with steroids and anesthetic at the greater occipital nerve	2 cm lateral and 2 cm inferior to the external occipital protuberance	8-48 weeks	Visual analog scale (VAS) + Global Perceived Effect (GPE)	Pain relief	Eight weeks after the initial treatment (T1), 66.7% of the patients were in the local injection group. Sixteen weeks after the initial treatment (T2), the success rate in the local injection group was 55.3%	8/15 (53.3%)	16 weeks	-
Lee et al. (2015) [20]	Observational study	Twenty-four consenting patients with chronic refractory neck pain and/or headache	Two atlantooccipital (AO) intra-articular injections of mixture of local anesthetic and steroid (1 week apart)	2 months	Pain drawings, visual analog scales (VASs) for pain	Pain relief	Fourteen patients (70%) had headache complaints. Before therapy, the mean VAS for headache was 5.6 (2.2); at one month, it was 1.9 (1.7) (p<0.01); and at two months, it was 0.6 (1.3) (p<0.01). A drop in VAS of more than 2 was observed across the board in 18 (90%) of the 20 individuals. Hundred percent of a 50% fall in their VAS score was attributed to a headache	18/20 (90%)	2 months	0/20 (0%)
Narouze et al. (2007) [21]	Retrospective study	Thirty-two patients with cervicogenic headache manifesting a clinical picture suggestive of atlantoaxial joint pain	Lateral atlantoaxial intra-articular steroid injection	6 months	Visual analog scale (VAS)	Pain relief	The mean pain scores were 1.9 (p<0.001)	26/32 (81.2%)	6 months	0/32 (0%)
Slipman et al. (2001) [22]	Retrospective study	Eighteen patients experiencing persistent daily headache symptoms for 3 months	Zygapophyseal intra-articular C2-3 joint injection	12-29 months (average 19 months)	Visual analog scale (VAS)	Pain relief	Every patient showed improvement after receiving a diagnostic intra-articular injection. Oral analgesics were effective in relieving 61% of patients with less than three headaches per week, and only 17% of patients reported no change in their symptoms	11/18 (61%)	19 months average	-

Radiofrequency Ablation

Radiofrequency ablation helps with cervicogenic headaches that emerge from abnormalities of the cervical spine typically structures innervated by the C1-C3 nerves [19-24]. Table 2 shows summarized characteristics of the studies included under the radiofrequency ablation intervention.

**Table 2:** Summarized characteristics of the studies included under the radiofrequency ablation intervention.

Author	Study design	Participants	Location of injection	Follow-up period	Pain measurement tool	Outcomes observed	Results	Pain relief (>50%)	Duration of pain improvement	Incidence of adverse effects
Haspeslagh et al. (2006) [19]	RCT	Fifteen patients with cervicogenic headaches undergoing radiofrequency treatments	Cervical facet joint + cervical dorsal root ganglion lesions	8-48 weeks	Visual analog scale (VAS) + Global Perceived Effect (GPE)	Pain relief	Eight weeks after the initial treatment (T1), 80% of the patients were in the RF group. Sixteen weeks after the initial treatment (T2), the success rate in the RF group was 66.7%	10/15 (66.7%)	16 weeks	-
Odonkor et al. (2017) [12]	Case reports	One female 27 years of age	Bilateral intra-articular radiofrequency ablation of the C1-C2 joint	2-12 weeks	Visual analog scale (VAS)	Function and pain relief	Treatment was effective and an 80% pain reduction was recorded. She evaluated her pain as being less intense by 8 and 12 weeks following the treatment, with week 12's score of 7 being the best	1 of 1 (100%)	8 weeks	-
Hamer and Purath (2014) [15]	Observational study	Forty patients with refractory cervicogenic headaches and or occipital neuralgia	Radiofrequency ablation of the C2 dorsal root ganglion and/or third occipital nerves	Six months to a year		Pain relief and adverse effects	Thirty-five percent of patients said their discomfort was completely gone, and 70% said it was at least 80% gone. Improvement has lasted on average for 22.35 weeks. Twelve to thirteen percent of cases involved complications. In the event that significant symptoms reappeared, 92.5% of patients said they would have the surgery done once more	22/40 (53%)	22.35 weeks	6/40 (12.5%)
Lee et al. (2020) [16]	Retrospective analysis	Forty-five electronic medical records of patients who underwent dorsal root ganglion (DRG) pulsed RFA	Radiofrequency ablation of the C2 dorsal root ganglion	6 months		Pain relief and adverse effects	Forty-five patients had C2 DRG pulsed RFA, and after 6 months, 40% of them (18/45) reported 50% pain reduction. Throughout the course of the investigation, there were no postoperative problems	18/45 (40%)	6 months	0/45 (0%)
Hu et al. (2022) [17]	A retrospective chart review	Forty-one patients who underwent CT-guided RFA	RFA of cervical intervertebral discs for cervicogenic headache (CEH)	6 months	Numeric rating scale (NRS)	Pain relief	RFA may be an effective treatment for patients with CEH, particularly for patients who have previously experienced definite pain reduction after C2 DRG block	28/41 (68%)	6 months	0/41 (0%)
Halim et al. (2010) [18]	Retrospective study	Eighty-six patients who had undergone lateral C1-2 joint PRF application for cervicogenic headache	Pulsed radiofrequency application into the lateral atlantoaxial (C1-2) Joint	2-12 months	Visual analog scale (VAS)	Pain relief	At 2 months, 6 months, and 1 year, the proportion of patients who reported ≥50% pain reduction was 50% (43/86), 50% (43/86), and 44.2% (38/86), respectively. A ≥50% pain alleviation at 2 months reliably predicted long-term pain relief at 6 months and 1 year (p<0.001)	43/86 (50%)	1 year	1/86 (0.0116%)

Meta-analysis

Radiofrequency Ablation: Pain Relief

**Figure 2:**
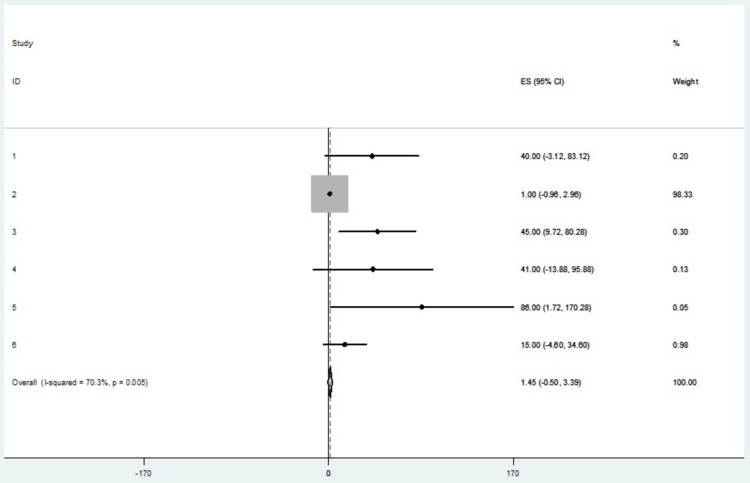
A forest plot for the outcome of pain relief in the RFA treatment intervention. RFA: radiofrequency ablation; ES: effect size

**Figure 3:**
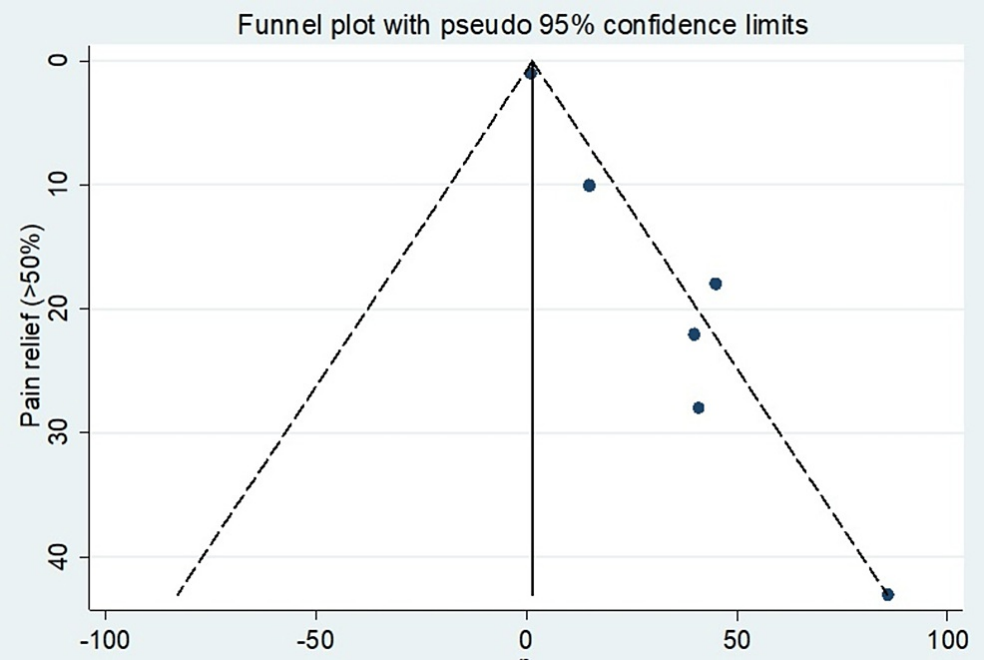
Funnel plot showing publication bias of the studies included.

The incidence of pain relief by a factor of >50% was the most observed outcome in this meta-analysis. All six studies reporting the use of RFA in managing cervicogenic headaches provided incident rates used to calculate effect sizes. Table 3 shows a summary incidence of pain relief in the studies included. Table 4 shows the mean of pain relief with respective confidence intervals. Table 5 shows the effect sizes at a 95% confidence interval. The forest plot and funnel plot depict this measure, which presents an overall random effects risk ratio of 1.45 (-0.50, 3.39) at a 95% confidence interval (Figures 2, 3). The test has a p-value of 0.145, which indicates a lack of significance. There was a moderately high level of heterogeneity, with the I-squared being 70.3%.

**Table 3:** Summary of studies included showing the incidence of pain relief.

Author	n	Pain relief (>50%)	Incidence percentage
Hamer and Purath (2014) [15]	40	22	53%
Odonkor et al. (2017) [12]	1	1	100%
Lee et al. (2020) [16]	45	18	40%
Hu et al. (2022) [17]	41	28	68%
Halim et al. (2010) [18]	86	43	50%
Haspeslagh et al. (2006) [19]	15	10	66%

**Table 4:** Mean pain relief of studies included. n (number of observations): 6

Variables	Mean	Standard error	95% Confidence interval
n	38.0000	11.9219	7.3536-68.6464
Pain relief	20.3333	5.9479	5.0437-35.6230
Incidence	0.62833	0.0857	0.4081-0.8485

**Table 5:** Radiofrequency ablation of pain relief effect sizes.

Study	Effect size (ES)	% Weight	95% Confidence interval
1	40.000	0.20	-3.119 to 83.119
2	01.000	98.33	-0.960 to 2.960
3	45.000	0.30	9.721 to 80.279
4	41.000	0.13	-13.879 to 95.879
5	86.000	0.05	1.722 to 170.278
6	15.000	0.98	-4.600 to 34.600
IV pooled ES	1.446	100.00	-0.498 to 3.389

Adverse effects of radiofrequency ablation

**Figure 4:**
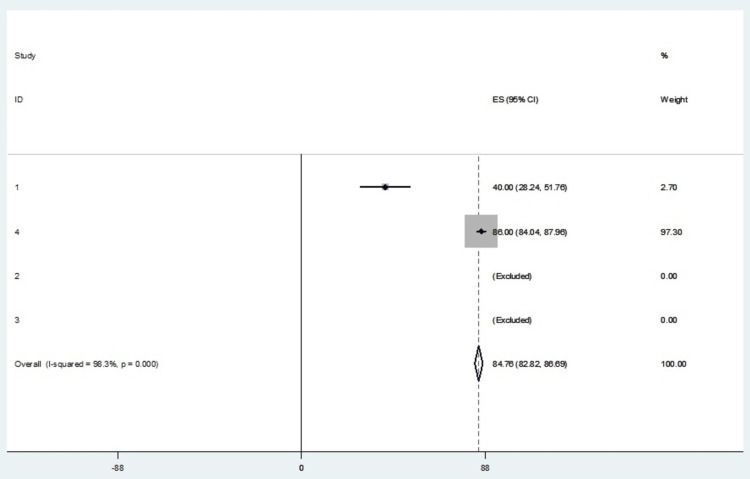
A forest plot for the outcome of adverse effects in the RFA treatment intervention. RFA: radiofrequency ablation; ES: effect size

**Figure 5:**
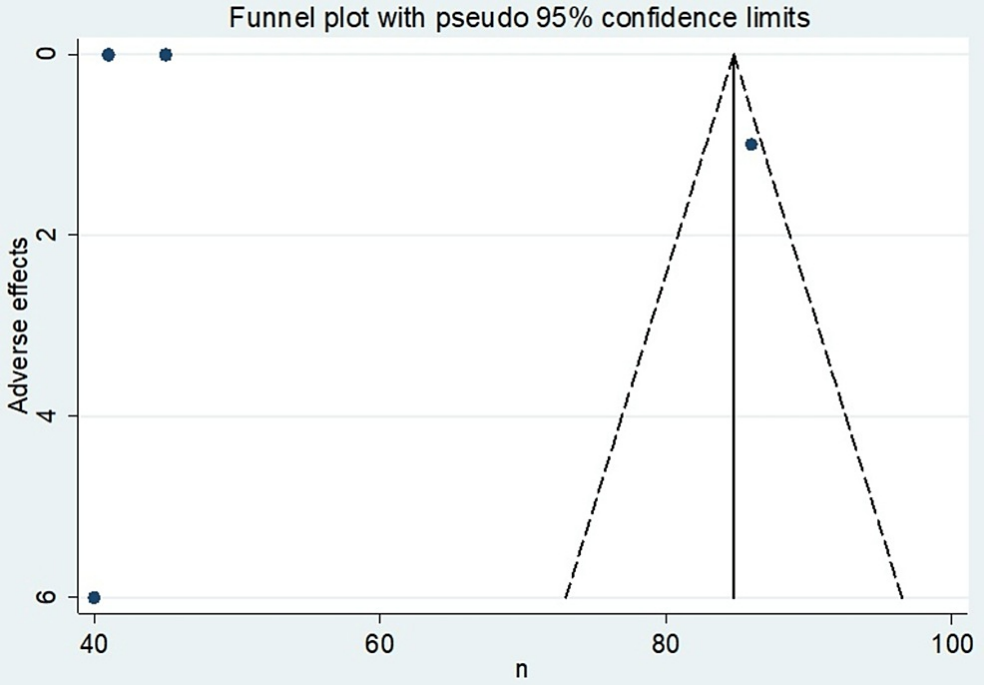
Funnel plot showing publication bias of the studies included.

The incidence of adverse effects was not common; however, it was reported in some of the studies. Table 6 shows the incidence percentage of adverse effects of radiofrequency ablation, while Table 7 depicts the mean adverse effect of radiofrequency ablation. Table 8 shows the effect sizes at a 95% confidence interval calculated on STATA. The forest plot in Figure 4 represents the effective measures. The meta-analysis finds an overall random effects risk ratio of 84.76 (82.82, 86.69) at a 95% confidence interval. The test has a p-value of 0.000 which indicates the existence of significance. There was a very high level of heterogeneity, with the I-squared being 98.3%. Figure 5 depicts the publication bias of the included studies.

**Table 6:** Incidence percentage of adverse effects of radiofrequency ablation.

Author	n	Pain relief (>50%)	Incidence percentage
Hamer and Purath (2014) [15]	40	22	12.50%
Lee et al. (2020) [16]	45	1	0%
Hu et al. (2022) [17]	41	18	0%
Halim et al. (2010) [18]	86	28	0.01%

**Table 7:** Mean adverse effect of radiofrequency ablation. n (number of observations): 4

Variables	Mean	Standard error	95% Confidence interval
n	53.0000	11.0529	17.8247 to 88.1753
Adverse effect	1.7500	1.4361	-2.8204 to 6.3204
Incidence	0.0325	0.0325	-0.0709 to 0.1359

**Table 8:** RFA adverse effects effect sizes. Heterogeneity chi-squared = 57.19 (d.f. = 1), p = 0.000, I-squared (variation in ES attributable to heterogeneity) = 98.3%; test of ES = 0, Z= 85.93, p = 0.00. RFA: radiofrequency ablation

Study	Effect size (ES)	% Weight	95% Confidence interval
1	40.000	2.70	28.240-51.760
4	86.000	97.30	84.040-87.960
2	(Excluded)	-	-
3	(Excluded)	-	-
IV pooled ES	84.757	100.00	82.823-86.690

Epidural steroid injection

Epidural Steroid Injection: Pain Relief

**Figure 6:**
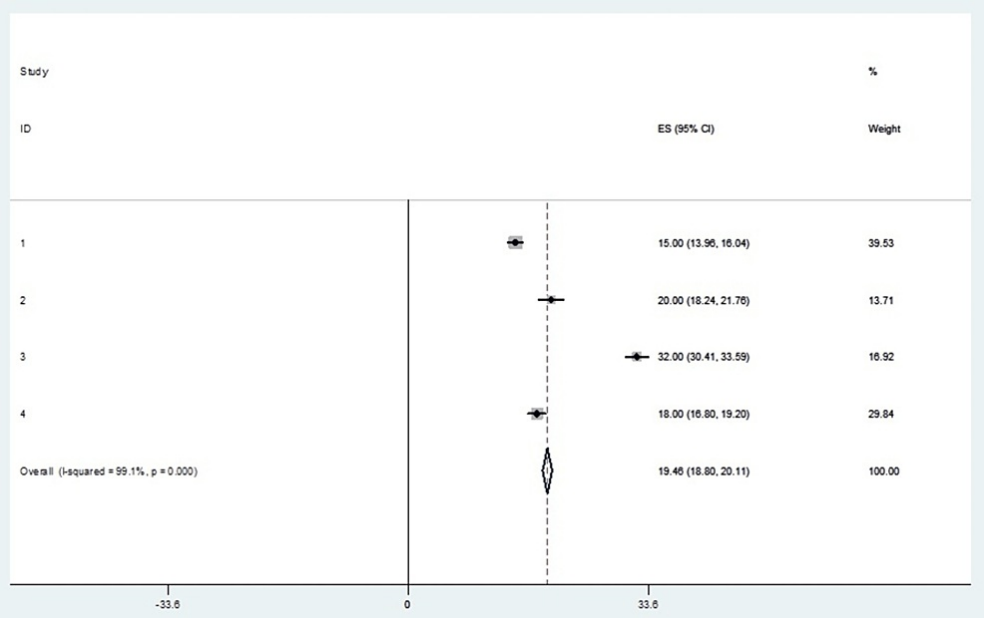
A forest plot for the outcome of pain relief in the ESI treatment intervention. ESI: epidural steroid injection; ES: effect size

**Figure 7:**
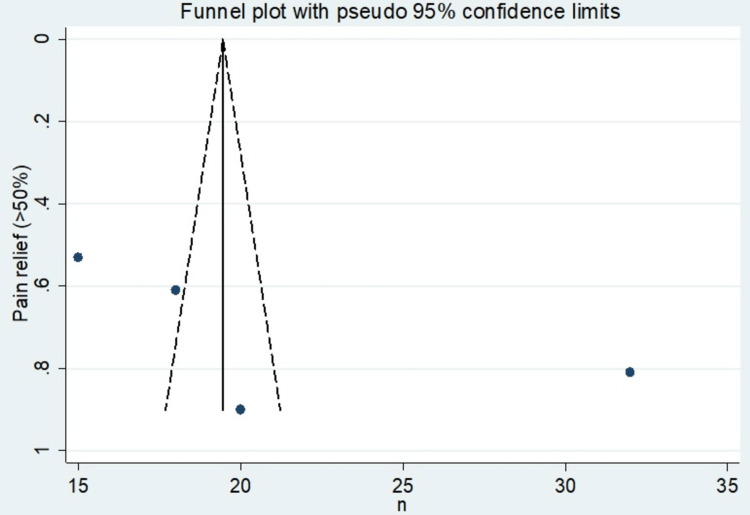
Funnel plot showing publication bias of the studies included.

The incidence of pain relief by a factor of >50% was the most observed outcome in this meta-analysis. All four studies reporting the use of ESI in the management of cervicogenic headaches provided incident rates used to calculate effect sizes. Table 9 shows the incidence percentage of pain relief at a 95% confidence interval. Table 10 shows the mean of epidural steroid injection of included studies. Table 11 shows the incidence effect sizes at a 95% confidence interval. The forest plot depicts this measure, which presents an overall random effects risk ratio of 19.46 (18.80, 20.11) at a 95% confidence interval (Figure 6). The test has a p-value of 0.000 which indicates the existence of significance. There was a very high level of heterogeneity, with the I-squared being 99.1%. Figure 7 depicts the publication bias of the included studies.

**Table 9:** Incidence percentage of pain relief for epidural steroid injection.

Author	n	Pain relief (>50%)	Incidence percentage
Haspeslagh et al. (2006) [19]	15	8	53.30%
Lee et al. (2020) [16]	20	18	90%
Narouze et al. (2007) [21]	32	26	81.20%
Slipman et al. (2001) [22]	18	11	61%

**Table 10:** Mean of epidural steroid injection studies included. n (number of observations): 4

Variables	Mean	Standard error	95% Confidence interval
n	21.2500	3.7277	9.3868-33.1132
Pain relief	0.7125	0.0859	0.4392-0.9858
Incidence	0.7125	0.0859	0.4392-0.9858

**Table 11:** Epidural steroid injection pain relief effect sizes. Heterogeneity chi-squared = 316.58 (d.f. = 3), p = 0.000, I-squared (variation in ES attributable to heterogeneity) = 99.1%; test of ES = 0, Z= 58.39, p = 0.00.

Study	Effect size (ES)	% Weight	95% Confidence interval
1	15.000	39.53	13.961-16.039
2	20.000	13.71	18.236-21.764
3	32.000	16.92	30.412-33.588
4	18.000	29.84	16.804-19.196
IV pooled ES	19.458	100.00	18.805-20.111

Adverse effects of epidural steroid injection

There were incidences of adverse effects reported by the studies included in this meta-analysis. However, only two studies mentioned this outcome, but both indicated zero major adverse effects events (Tables 12, 13).

**Table 12:** Distribution of adverse effects in radio frequency ablation.

Author	n	Adverse effects	Incidence percentage
Lee et al. (2020) [16]	20	0	0%
Narouze et al. (2007) [21]	32	0	0%

**Table 13:** Zero incidence rates of adverse effects during management with ESI. n (number of observations): 2 ESI: epidural steroid injection

Variables	Mean	Standard error	95% Confidence interval
n	26.000	6.000	-50.2372-102.2372
Pain relief	0.000	0.000	-
Incidence	0.000	0.000	-

Discussion

The studies fundamentally define cervicogenic headaches as the type of headaches that emerge from abnormalities of the cervical spine or the neck's soft tissue. Typically, structures innervated by the C1-C3 nerves can be responsible for cervicogenic headaches because of the physiological linkage of the top section of the cervical spinal cord and trigeminal nerves [23]. Cervical pain is a characteristic symptom of a wide range of headaches; hence, it does not conclusively establish the diagnosis of cervicogenic headache [16-20]. Cervicogenic headaches are principally unilateral and episodic with varying severity emanating from the neck to the ocular, occipital, frontal, and temporal region in a C-shaped configuration. Clinically characterized by head pains during neck movement or prolonged maintenance of a single posture, cervicogenic headaches are associated with a limited range of neck motion and pain from the neck radiating to the posterior and anterior regions of the head [7,19,23]. The treatment of cervicogenic headaches has taken an integrative approach that combines physical therapy, anesthetic, and surgical approaches. Some of the interventions include radiofrequency ablation and epidural steroid injection. The efficacy of RFA and ESI differ as established by several characteristics, including pain intensity, duration, and complications. Comparison of RFA and local injections of steroids establishes insignificant differences between the two interventions [19].

Radiofrequency ablation

Radiofrequency procedures have increasingly gained prevalence as the healthcare industry seeks minimally invasive procedures of complex spinal surgery. Attempts to develop minimally invasive techniques have inspired the expansion of the intersection between RF and the nervous system [24]. In addition, advances in pain care inspired the use of RF in its interventions. Radiofrequency procedures typically produce highly efficient site-specific pain relief interventions; hence, they can be exploited in treating cervicogenic headaches.

Pain Intensity

Hamer and Purath emphasize radiofrequency ablation’s highly effective nature in reducing pain intensity [15]. They established that, in most instances, the patients who underwent the RFA intervention typically experienced a high percentage of pain relief, with some of the patients even experiencing complete pain relief after the intervention [24,25]. The integration of RFA with technologies such as computed tomography has essentially increased the intervention's impact on pain intensity as the source of cervicogenic headache can be easily pinpointed, and completely ablated [12,17]. Generally, the efficiency of RFA as an interventional treatment for cervicogenic headache is increasingly becoming more effective, depending on the technology utilized in guiding the process. Lee et al. argue that fluoroscopy-guided C2 dorsal root ganglion is a highly effective intervention for cervicogenic headaches [16].

Pain Duration

According to Hamer and Purath, the average duration for improvement of cervicogenic headache is twenty-two-and-a-half weeks [15]. The duration required for an individual to experience pain relief of more than 50% can be reliably established in six months to one year [16-18].

Complication

While RFA is highly effective in the reduction of pain intensity, it has a considerably high complication rate, which falls between 12% and 13% [15]. Despite the high complication rate, most of the patients who have experienced RFA prefer to repeat the procedure in case of recurrence. Hu et al. record a considerably lower complication rate, highlighting the role of technological advancement in the evolution and increasing efficacy of RFA as an intervention for CHA [17]. According to Lee et al., there were no complications for patients who underwent fluoroscopy-guided C2 dorsal root ganglion, which insinuates the complications in RFA can be essentially reduced through imaging guidance during the ablation [16].

Epidural injections steroid injection

Pain Intensity

Lee et al. argue that the intra-articular injection is an effective intervention in the short-term management of pain [20]. Moreover, it is highly effective in handling some defining characteristics of cervicogenic headaches, such as the neck disability index. Slipman et al. argue that injections are highly effective in reducing the intensity of cervicogenic headaches, particularly in the short term [22].

Pain Duration

According to Lee et al., the neck disability index of the patients is considered to reduce significantly in two months, which offers insight into the duration of pain [20]. The pain duration is considerably lower compared to the RFA. However, pain persists after the epidural steroid injection, demanding the consumption of oral analgesics to deal with them; hence, the duration of the injection cannot be established [22].

Complication Rate

According to Slipman et al. more than half of the patients who had experienced injections experienced three headaches per week, which were relieved by oral analgesics [22]. While the weekly headaches can be considered complications of the treatment, it could mean the oral analgesics are part of an integrative approach to treating cervicogenic headaches [19,21]. Therefore, at the same time, the injections are effective in treating cervicogenic headaches and are supported by other medical treatments [26-28].

Study limitations

The study cannot arrive at conclusive decisions because few studies explore the efficiency of RFA and ESI in treating cervicogenic headaches. The included studies generalize pain and injection, hence lacking the specificity necessary to arrive at distinct results. The difficulty in diagnosing cervicogenic headaches typically contributes to the study's limitations, as the different headaches fundamentally share characteristics and symptoms such as cervical pain. The difficulty in diagnosis essentially results in few participants in the research for CHA interventions. Moreover, the study was fundamentally limited by the subjectivity of pain, which demands that the research depends on the participants' feelings. The difficulty in diagnosis fundamentally resulted in a small sample, affecting the accuracy of the studies included in the research.

## Conclusions

Cervicogenic headaches are chiefly unilateral headaches but might be felt bilaterally in severe cases, originating from the neck tissue or spinal cord. Typically, cervicogenic headaches have localized, episodic pain projecting from the neck to the rest of the head, including ocular, occipital, frontal, and temporal regions in a C-shaped configuration. Cervicogenic headaches have a high neck disability index, characterized by a limited range of neck motion and pain from the neck radiating to the posterior and anterior regions of the head. Traditionally, there are different strategies for treating cervicogenic headaches classified into three main categories as follows: physical therapy, anesthetics, and surgical approaches. Of these treatments, some of the interventions include radiofrequency ablation and epidural steroid injection. Generally, these strategies can be implemented collaboratively, as in the case of epidural steroid injections and oral analgesics, or independently, like in the case of RFAs. The efficacy of RFA and ESI differ as established by several parameters, including pain intensity, duration, and complications. Typically, both interventions are effective in the reduction of pain intensity; however, their complication rates and pain duration are considerably different. With the epidural steroid injection, the headaches can still recur weekly, demanding the use of oral analgesics to deal with them. On the other hand, RFA has a low complication rate, particularly when guided through fluoroscopy or computed tomography. Generally, with improving guidance from imaging technologies, RFA has the potential to be the most effective intervention due to the increased accuracy, which can increase the reduction of pain duration and intensity.
